# Comparison of Breast Cancer and Cervical Cancer in Uzbekistan and Korea: The First Report of The Uzbekistan–Korea Oncology Consortium

**DOI:** 10.3390/medicina58101428

**Published:** 2022-10-10

**Authors:** Chai Hong Rim, Won Jae Lee, Bekhzood Musaev, Ten Yakov Volichevich, Ziyayev Yakhyo Pazlitdinovich, Hye Yoon Lee, Tillysshaykhov Mirzagaleb Nigmatovich, Jae Suk Rim

**Affiliations:** 1 Department of Radiation Oncology, Korea University Ansan Hospital, Korea University College of Medicine, Seoul 02841, Korea; 2 Department of Healthcare Management, Gachon University, Seongnam-si 13120, Korea; 3 Minister, Ministry of Health of the Republic of Uzbekistan, Tashkent 100086, Uzbekistan; 4 Republican Specialized Scientific Practical-Medical Center of Oncology and Radiology, Farobiy Street 383, Tashkent 100179, Uzbekistan; 5 Division of Breast and Endocrine Surgery, Department of Surgery, Korea University Ansan Hospital, Korea University College of Medicine, Seoul 02841, Korea; 6 Department of Oral & Maxillofacial Surgery, Korea University College of Medicine, Seoul 08308, Korea

**Keywords:** Uzbekistan, cancer, treatment, screening, prevention

## Abstract

In general, as a country’s economy, education level, and life expectancy increase, the incidence of cancer increases. This is because the peak incidence of cancer occurs in individuals in their 70s and 80s, and the health proportion of non-communicable diseases increases with the development of the living environment. Changes in diet, lifestyle and enhanced methods of detection contribute to an increase in cancer incidence as well. Recently, Uzbekistan has grown rapidly, and its incidence of cancer is also increasing. In the health management of cancer, not only treatment but also the identification and prevention of causes and effective screening should be considered. South Korea has a common ethnicity with Uzbekistan and has successfully performed national screening for seven major cancers over the past 20 years. The 5-year survival rate after cancer diagnosis in Korea was only 42.9% 20 years ago, but recently it has improved to 70.7%. We formed an advisory consortium in which oncologists from Uzbekistan and Korea could cooperate for cancer management in Uzbekistan. This advisory consortium intends to present the necessary considerations and recommendations for cancer management in Uzbekistan by examining the literature and cancer statistics of Uzbekistan and South Korea. In addition to the overall analysis, we identified and reviewed the major cancers with high morbidity in three categories in Uzbekistan: gynecological cancer (breast and cervical cancer), cancer common in men (lung and liver cancer), and gastrointestinal cancer (stomach and colorectal cancer). This review covers the general cancer statistics of Uzbekistan and a detailed review of gynecological cancer between two countries, and relevant recommendations.

## 1. Introduction

As the human developmental index of society (the index of the UN [1] which comprehensively evaluates life expectancy, health, education, economic level, etc.) increases, cancer incidence tends to increase. Cancer generally increases after the age of 65 and peaks in the 70s and 80s [2]. As education and economic standards improve, deaths from communicable diseases such as infectious diseases decrease, and deaths from non-communicable diseases (cancer, hypertension, diabetes, etc.) increase [3]. Since the diagnosis and treatment of cancer require a certain level of scientific technology and economy, the detection rate of cancer itself increases side by side with society’s development.

Uzbekistan has grown rapidly economically and socially recently. With this growth, cancer incidence has also been increasing [4]. To effectively respond to the increasing health burden, the Ministry of Health of Uzbekistan formed a consortium of surgical oncologists, radiation oncologists from Tashkent National Cancer Center, and advisors from South Korea, including radiation oncologists, surgical oncologists, and medical informatics specialists. Our consortium will identify the characteristics of cancer burden in Uzbekistan and suggest treatment and prevention strategies. The cancer data of South Korea will be introduced as a control. Although cancer has been the top cause of death in South Korea, its management has been effective with nationwide efforts. As a result, the 5-year cancer survival rate was 42.9% between 1993 and 1995, improving significantly to 70.7% between 2015 and 2019 [5]. For the last two decades, the Korean government has been conducting nationwide free screenings for seven types of cancer: stomach cancer, liver cancer, lung cancer, colorectal cancer, breast cancer, and cervical cancer, which has contributed to improving the survival rate due to the early detection of cancer.

We would like to present recommendations for cancer management in Uzbekistan based on the following information and other published literature: GLOBOCAN 2020 by IARC [6,7,8,9], the WHO’s Cancer Country Profile 2020 [10,11], imPACT review report of Uzbekistan co-worked by IAEA, IARC, and the WHO [12], and the National Cancer Information Center of Korea [5]. We searched for reference studies on cancer prevention and treatment in Uzbekistan among studies published up to 15 May 2022 in Pubmed, Embase (using the keywords of cancer prevention, cancer treatment, and Uzbekistan), and Yahoo.com, including Russian literature. A search engine was used. The target readers for this study are oncologists, healthcare providers who are not oncologists, and health policymakers. In addition to the overall oncological literature analysis, we analyzed the major cancers of Uzbekistan with high mortality and morbidity in three categories: gynecological cancer (breast cancer and cervical cancer), cancer common in men (lung and liver cancer), and gastrointestinal cancer (stomach and colorectal cancer).

In this review, we will analyze the causes, frequency, and types of cancer in Uzbekistan; a detailed review of gynecological cancer and related recommendations will be discussed in this review.

### Cancer Statistics of Uzbekistan from Global Perspective

According to a 2020 report by IARC, the age-standardized incidence of cancer per 100,000 people in Uzbekistan is 108.1. The corresponding rate in South Korea is 242.7 [6,7,8,9]. Incidence is 102.5 in South-central Asia (where Uzbekistan is), 111.1 to 130.2 in Sub-Saharan African regions, 217.2 in East Asia, 325 in Western Europe, 360.7 in Northern America, and 447.6 in Australia and New Zealand (the highest among these regions). Since HDI in Uzbekistan has been steadily increasing in recent decades, the national cancer incidence is expected to continue to increase. Regarding age-standardized mortality per 100,000 people, this is 72.4 and 75.5 in Uzbekistan and South Korea, respectively. Although the incidence of cancer is much higher in South Korea, the amount of mortality seems similar. Regionally, the corresponding values are 67.0 in South-central Asia, 78.4 to 9.1 in Sub-Saharan African regions, 123.2 in Eastern Asia, 103.3 in Western Europe, 87.1 in Northern America, and 85.8 in Australia and New Zealand. As a rough indicator to understand the status of cancer management, we calculated the rates of mortality per incidence (per 100,000): they are 67% and 31.1% in Uzbekistan and South Korea, respectively, and 65.4%, 69.2–70.6%, 56.7%, 31.8%, 24.1% and 19.2% in South-Central Asia, Sub-Saharan African regions, East Asia, Western Europe, Northern America, and Australia and New Zealand (Table 1). To summarize, the overall cancer incidence in developed countries is higher than in developing countries, but the incidence per mortality is considered to be lower in countries with sufficient cancer treatment and diagnostic infrastructure.

Regarding cancer specificity, lung cancer is the leading cause of male cancer death in Central Asia, East Asia, and most of the Northern Hemisphere [6]. In some countries (Iran, Afghanistan, Tajikistan), stomach cancer ranks first in mortality, and lip and oral cavity cancer is the leading cause of cancer death in India. Breast cancer is the leading cause of cancer death among women in most Central Asian countries, including Uzbekistan. In East Asia, including Korea and China, lung cancer is the leading cause of death for both men and women.

**Figure 1:**
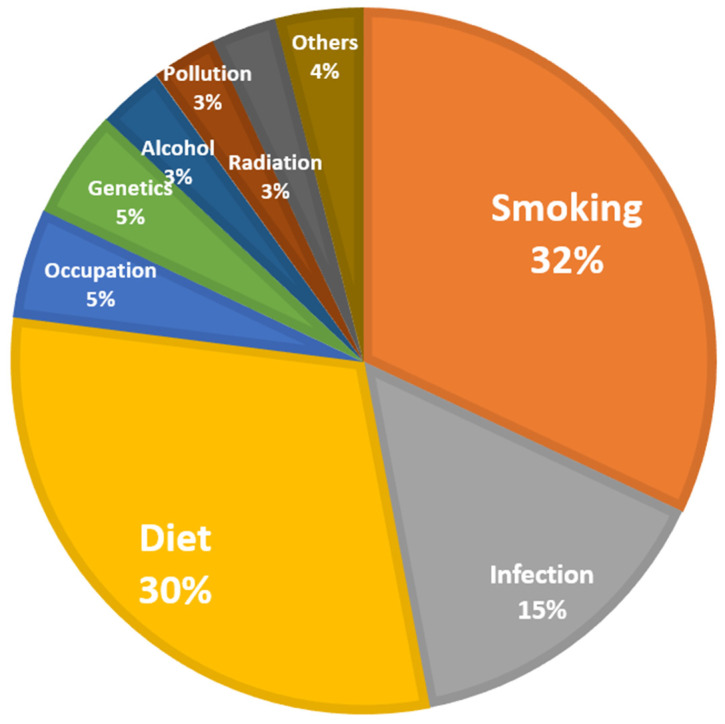
Summary of causes of cancer. Data source: World Cancer Report by IARC, 2008 [13]. Pie chart drawn by authors.

According to the IARC cancer report in 2008, approximately one-third of cancer was caused by smoking, one-third food-related, and one-third by other causes (e.g., infection, occupation, hereditary, alcohol, pollution, and radiation) (Figure 1) [13]. Referring to the WHO cancer country profile in 2020, the factors expected to affect the incidence of cancer in Uzbekistan are infection (21.3%), tobacco (13.8%), ultraviolet rays (9.7%), alcohol (7.8%), and obesity (3%) [10]. The causes of cancer in Uzbekistan are high in terms of the proportion of infection and ultraviolet rays, and the proportion of smoking is small compared to the common causes of cancer. However, since it has not been investigated internally, and it is not specified which type of cancer and which causes are related, domestic investigation seems necessary.

**Figure 2:**
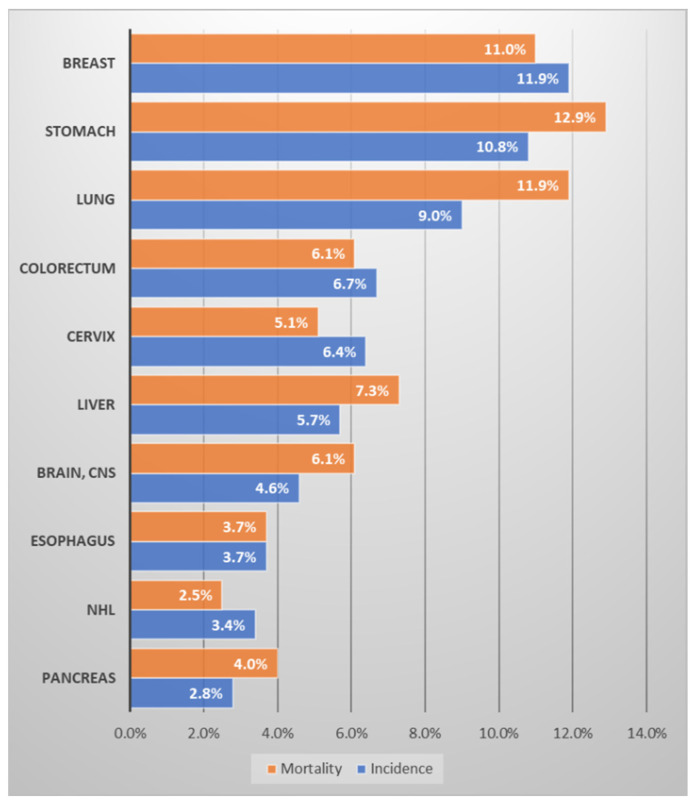
Ranking of the frequency and mortality of major cancers in Uzbekistan. Data source: Cancer country profile, WHO, 2020; Global Health Observatory, WHO, 2016. Figure drawn by authors [10,14].

**Figure 3:**
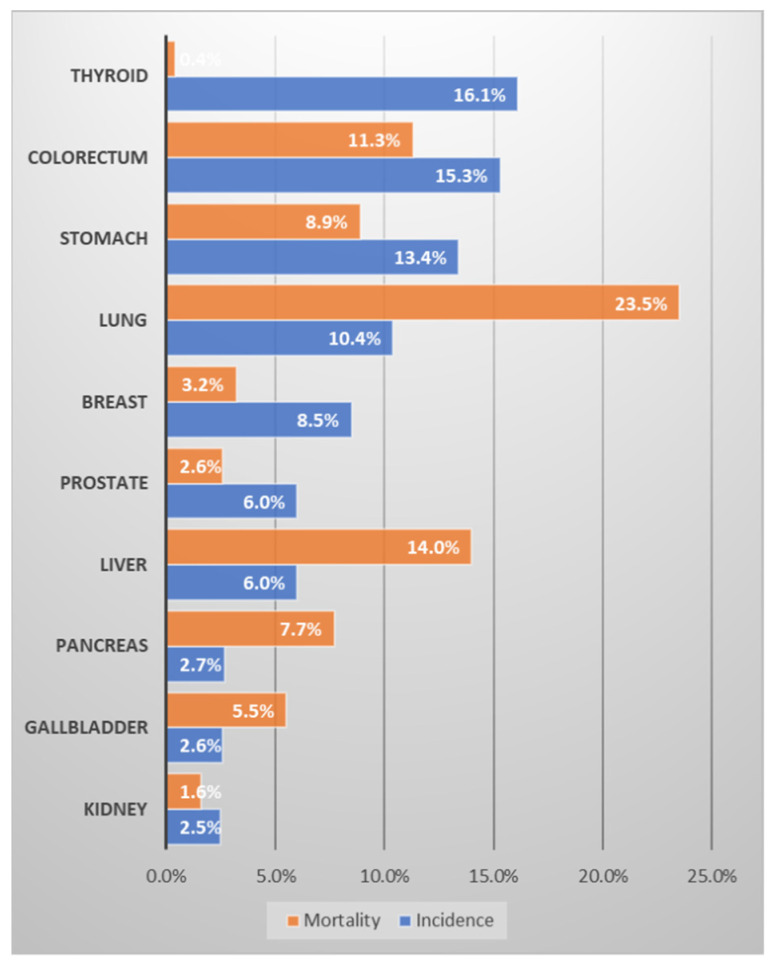
Ranking of the frequency and mortality of major cancers in Korea. Data source: Cancer country profile, WHO, 2020; Global Health Observatory, WHO, 2016. Figure drawn by authors [11,14].

In terms of cancer prevalence in Uzbekistan, breast cancer (11.9%) is followed by stomach cancer (10.8%), and lung cancer (9%), and in terms of mortality, stomach cancer (12.9%) is followed by lung cancer (11.9%) and breast cancer (11%) (Figure 2) [10,14]. The three major cancers, breast, stomach, and lung, account for about one-third of cancer incidence and mortality in Uzbekistan. Accompanying cervical cancer, colorectal cancer, and liver cancer, six major cancers account for little more than half of cancer incidence and mortality. Of note is that although breast cancer occurs only in women, it has the greatest incidence and third highest mortality. According to the same data source regarding South Korea (Figure 3 [11]), the highest incidence was occupied by thyroid cancer; however, since it has a very low fatality rate (<1~2%), it might be inappropriate to define that as a major cancer causing health burden. Excluding thyroid cancer, the most frequent cancers were colorectal cancer (15.3%), stomach cancer (13.4%), and lung cancer (10.4%), in that order. In terms of mortality, lung cancer was overwhelming (23.5%), followed by liver cancer (14%) and colorectal cancer (11.3%). Pancreatic cancer has a frequency of only 2.7%, but its mortality rate is significant at 7.7%. Considering that breast cancer occurs almost exclusively in women, its frequency is considerable (8.5%), but it is relatively insignificant as a cause of mortality (3.2%).

In summary, although the cancer incidence in Uzbekistan is low internationally, the mortality is high compared to the incidence. Compared to Korea, the incidence is about 40% (242.7 vs. 108.1 cases per 100,000 population), but the mortality rate is almost the same (72.4 vs. 75.7). Therefore, the incidence per mortality is as different as 67% vs. 31%. Therefore, it is necessary to encourage cancer screening to lower the stage at diagnosis and reinforce the infrastructure for treatment. Infection and ultraviolet rays are prominent causes of cancer in Uzbekistan, and the proportion of smoking is small compared to the common causes of cancer. In terms of cancer incidence and mortality, breast cancer, stomach cancer, lung cancer (first major cancer), cervical cancer, colorectal cancer, and liver cancer (second major cancers) account for just over a half. Although breast cancer mostly occurs in women, it ranks third in mortality with the highest prevalence in the entire population.

## 2. Breast Cancer

### 2.1. Incidence and Mortality

Although breast cancer occurs in women (99% of the disease occurs in women [15]), in Uzbekistan, it has the highest incidence rate and a significantly high mortality rate overall (Figure 2) [10,14]. Compared with South Korea, the incidence of breast cancer in Uzbekistan is less significant, but mortality is high. In Uzbekistan, breast cancer incidence and mortality per 100,000 population members are 26.4 and 12.8, respectively, yielding a 48.5% mortality/incidence ratio in 2020. In comparison, in South Korea, the age-standardized incidence and mortality per 100,000 population were 64.2 and 6.4, respectively, yielding a 9.9% mortality/incidence ratio [6]. From an international perspective, breast cancer is more common in first-world countries [6]; according to an American study, breast cancer is more prevalent in White and Black people than in Asian and Hispanic people [16,17]. In regions including Western Europe, Australia, and North America, the standardized incidence rate is close to 90 per 100,000, but the mortality rate is very low at 12–16 per 100,000. On the other hand, in Central Asia, to which Uzbekistan belongs, the age-standardized incidence per 100,000 is as low as 26, but the mortality is 13, similar to mortality in Western Europe, Australia, and North America (Table 2). Although the incidence of breast cancer in Uzbekistan is relatively low, the amount of mortality is similar to that of Western countries. 

**Figure 4:**
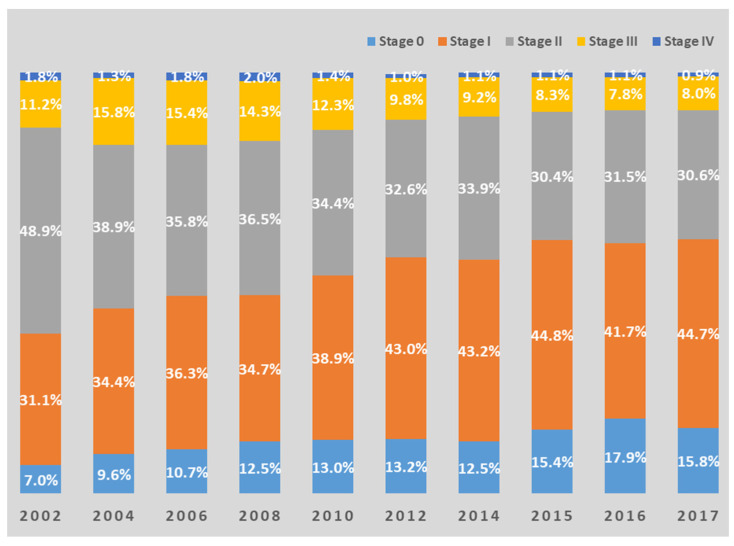
Changes in distribution by stage of breast cancer in Korea. Data Source: Korean Breast Cancer Society [18]. Bar charts are redrawn by authors.

According to the data of the Korean Breast Cancer Society, from 2002 to 2017, the number of breast cancers detected at stage 0 or 1 increased significantly (from 38% to 60%). On the other hand, breast cancer detected at stage 3 or higher decreased from 17% to 9% (Figure 4) [18]. The commonest stage was stage I (43.2%), followed by stage II (27.3%) in 2018 [19]. The 5-year survival rate of breast cancer patients in Korea steadily increased from 79.3% in 1993–1995 to 93.6% in 2015–2019 [5]. In comparison, according to the imPACT report of Uzbekistan by IARC, the commonest stages of breast cancer are 2 and 3, and the overall 5-year survival rate is 42.5% [12]. If the disease is detected earlier, the possibility of cure and longer survival increases, and it is advantageous to utilize health resources because the treatment of early cancer is simpler. Therefore, in Uzbekistan, it is necessary to screen breast cancer at an early stage through an active national examination program to improve treatment efficiency.

### 2.2. Benefits of Screening

Mammography or ultrasound are commonly used tools for screening breast cancer. In South Korea, free mammography is performed every two years for women over 40 years of age as a national screening program. According to a recent meta-analysis, early breast cancer screening using mammography was able to reduce cancer mortality from breast cancer by 19%. Furthermore, the total number of surgeries were also increased by 31%, which means more patients in the screening arm were treated with curable options [20]. A comparative study conducted in Norway investigating the efficacy of the national breast cancer screening program showed that the 5-year breast cancer survival rate increased from 76% to 91% [21]. Since these are relatively inexpensive imaging modalities, as compared to equipment such as computed tomography or magnetic resonance imaging, nationwide screening using mammography or sonography is recommended in Uzbekistan.

Since the breast is an organ located on the outside of the body, breast cancer is a disease that can be screened through clinical or self-examination. The benefit of clinical or self-examination is especially significant in developing countries, where imaging screening is not commonly performed, and the majority of breast cancers are diagnosed at advanced stages [22]. For the details of examination programs and maneuvers, it is recommended that domestic breast cancer surgeons of Uzbekistan distribute guidelines considering the culture of Uzbekistan. Some researchers have reported that women of Islamic culture might have limited direct information access outside their home, and they could be reticent about asking related questions to healthcare providers [23]. Any religious aspects that hinder the screening should be carefully addressed; for example, people might believe cancer is a religious punishment, and screening is a method to avoid it [24]. Furthermore, there may be a sense of reluctance for women to be examined by male medical staff. It would be ideal if enough female physicians could be trained in the long term, but it is recommended in the short term to train and utilize nurses who specialize in gynecologic cancer screening.

### 2.3. Identify the Cause of Breast Cancer

Known high-risk causes of breast cancer include an early age of menarche (<11), a late age of menopause (>54), late age at first pregnancy (>40), and family history (breast cancer in a first-degree relative) (relative risk ratio 2–3). High intake of saturated fat, obesity, excessive alcohol intake, use of oral contraceptives, smoking, and hormone therapy are also known risk factors (relative risk ratio 1.3–2) [25,26]. Although the prevalence of breast cancer is high in Uzbekistan, it was difficult to find domestic data investigating the causes of breast cancer. Due to the dynamics of the Islamic culture, risk factors such as alcohol and tobacco seem to affect women less than men. According to the WHO tobacco control fact sheet, the smoking population in Uzbekistan was 14.4% as of 2017, which was a moderate level globally, but comprised 26.8% men and 1.4% women [27]. Breastfeeding can reduce the risk of breast cancer by up to 39%, according to a recent meta-analysis [28]. Therefore, whole breastfeeding (complete nutrition intake only through breastfeeding) helps prevent breast cancer until about 6 months after childbirth [29]. For people with high-risk age factors, for those with breast cancer family history or no experience of childbirth, more active clinical screening should be recommended. 

### 2.4. Advice on Treatment

A surgical approach is usually the mainstay of treatment for breast cancer. Regarding locoregional treatment, in brief, radiotherapy is performed after breast-conserving surgery in stage II or lower-stage disease, and total mastectomy and radiotherapy are performed optionally at stage III or some stage II cases [30,31]. However, in countries where radiation therapy is not popularized, total mastectomy is performed even for early breast cancer. According to a recent survey by IARC, 70% of breast cancer surgeries performed in Uzbekistan are mastectomy and 30% are breast-conserving surgery [12]. Among breast cancer surgeries performed in South Korea, the proportion of breast-conserving surgery was 66% in 2018 [19]. The high rate of breast-conserving surgery in Uzbekistan may be due to the high stage of diagnosis and the lack of popularization of radiation therapy. Therefore, it is necessary to reduce the rate of mastectomy and increase breast-conserving surgery through the popularization of radiation therapy availability and screening.

Considering that the most common stages of breast cancer diagnosed in Uzbekistan are stage II and III, most breast cancer patients are subject to chemotherapy or hormone therapy before and after surgery [30,31]. The role of systemic therapy using targeted anticancer drugs or hormones is commonly used, as well as classical anticancer drugs. Unlike surgery or radiation therapy, drug administration might require highly skilled physicians and up-to-date equipment, but it is economically burdensome because most drugs must be imported. For example, trastuzumab costs range from USD 2761 to USD 14,261 for weekly use, which represents a concern in developing countries [32]. According to a recent study in Russia, about 70% of the cost of breast cancer treatment was related to systemic therapy [33]. Financial allotment from the government level and cooperation with major drug companies are necessary. Down-staging at diagnosis via screening can reduce the economic burden as the necessity of drug administration is reduced.

### 2.5. Summary and Suggestions

The prevalence and mortality of breast cancer in Uzbekistan are very high, so urgent health intervention is required. Since the high mortality compared to the incidence suggests that a significant portion of patients are detected at an advanced stage, screening at a national level using mammography or ultrasound is deemed necessary. Education on self-examination and the administration of routine clinical exams could also be beneficial. It is necessary to suppress the increase by identifying the specific cause of breast cancer in Uzbekistan. In terms of treatment, radiotherapy availability should be enhanced to increase the application of breast-conserving surgery. Economic considerations from the government for the active use of novel targeted or hormone drugs are needed.

## 3. Cervical Cancer

### 3.1. Incidence and Mortality

According to the WHO cancer country profile in 2020 (Figure 2), the prevalence of cervical cancer in Uzbekistan is 6.4%, and it is the fifth most common cancer. However, since cervical cancer only affects women, the rate of 6.4% is not negligible. Regarding prevalence, cervical cancers are the second most common cancer (10.6%), following breast cancer (24.9%), among female cancers [10]. Furthermore, the actual incidence may be higher than that reported in Uzbekistan. Women in the Islamic culture may believe that the detection of cervical cancer is related to an extra-marital relationship, or they may be reluctant to mention the genital organ, thereby hiding symptoms or being reluctant to have screenings. Additionally, social interactions with people outside the family (e.g., healthcare providers) tend to be limited [34]. In South Korea, it was one of the top three female cancers before 2000, but now ranks 10th and accounts for 2.6% of all female cancers [5].

The incidence of cervical cancer is closely related to the HDI of the region, as well as vaccination and screening programs [7]. The incidence in Central Asia is in the middle among global regions, and African countries, Southeast Asia, and South America are located above this [6]. In Uzbekistan, cervical cancer incidence and mortality per 100,000 population members were 11 and 6.7, respectively, yielding a 61% mortality/incidence ratio. In comparison, age-standardized incidence and mortality (per 100,000 population) were 8.1 and 1.8, respectively, yielding a 22.2% mortality/incidence ratio [6] (Table 3). We could not find the 5-year survival rate for cervical cancer in Uzbekistan. In South Korea, the 5-year overall survival rate was 80.5% from 2015 to 2019 [5]. About 55% of cervical cancers were diagnosed at stages I-II from 2010 to 2020 in Uzbekistan [35]; in comparison, stage I-II comprised 86.3% of all cervical cancer data in Korea from 1999 to 2004 [36].

In summary, the prevalence of cervical cancer in Uzbekistan is considerable. However, early detection through screening and effective treatment are required due to the high mortality rate compared to the prevalence.

### 3.2. Cause of Cervical Cancer 

Cervical cancer is related to various clinical and social factors. Aggarwal [37] reported that smoking history (relative risk: 2–4), insufficient intake of folic acid, carotene, and vitamin C (2–3), a low socio-economic class (1.5), low education level (2–3), multiparity (2–4), and lack of screening (2–6) were described as major risk factors for cervical cancer. According to ICO/IARC data in 2021, the smoking rate among women in Uzbekistan was 1.3%, and contraceptive use was 2.3%, neither of which was high. The total fertility rate (live births per woman) was 2.2, similar to the global average [38]. The most important cause of invasive cervical cancer is the human papilloma virus (HPV); HPV is found in about 99.7% of cervical cancer patients [39]. An early onset of sexual activity (relative risk: 2–4), multiple partners (2–5), a history of sexual disease (4–10), early age at first birth (2–4), and immunosuppressive status (5.7) have been reported as major causes of cervical cancer [37] which are directly or indirectly related to HPV.

### 3.3. HPV and Vaccination

There is a social perception that HPV infection is associated with promiscuous sexual life, which can result in hiding the infection status or associated disease. This delays the detection of diseases such as cervical cancer and increases mortality. Contrary to popular belief, HPV infection is highly contagious and can be transmitted even through skin-to-skin contact, and it is difficult to clearly define the route of infection in most cases. In fact, most people who live a normal sexual life are infected with HPV at least once in their lifetime [40]. Therefore, it is necessary to educate the public that it is very common (more than half of the global population is infected), that it is virtually impossible to fully prevent while socializing, and that it is not necessarily related to sexual life. In addition, cervical cancer can be detected with symptoms occurring in the early–mid stage; cancers having such symptoms are more likely to be detected and treated at an early stage. A typical symptom of cervical cancer is vaginal bleeding after sexual intercourse, or regardless of sexual intercourse, which may cause severe or irregular bleeding [41]. Once again, it is necessary to educate people that cervical cancer or HPV may not be related to promiscuous sexual life to prevent disease progression by hiding symptoms and infection status.

About 70% of cervical cancers are related to HPV 16 and 18, and 20% are related to 31, 33, 45, 51, 58. Since 90% of anal, vaginal, and head and neck cancers are also related to HPV 16 and 18, the use of HPV vaccines is also related to the prevention of these cancers [42]. There are three best-known HPV vaccines. HPV quadrivalent vaccine (trade name: Gadasil) covers HPV 6, 11, 16, and 18; HPV 9-valent vaccine (trade name: Gadasil 9) covers HPV 6, 11, 16, and 18, as well as 31, 33, 45, 52, 58; and HPV bivalent vaccine (trade name: Cervarix) covers only 16 and 18. Generally, the type with a wide range of virus coverage is more expensive.

The efficacy of all three vaccines is very high. The effectiveness of preventing cervical cancer or precancerous lesions (lesions that have not infiltrated or metastasized but can progress to invasive cancer) was 97–100% in the HPV-naïve population and 44–61% in the total population [43,44,45,46]. The efficacy of preventing vaginal cancer or its precancerous lesions was similar. According to the Advisory Committee on Immunization Practices of the US [47], vaccination can be administered from 9 years and is recommended for 11–12 years of age. Vaccination for men as well as women is effective. People between the ages of 13 and 26 who have never been vaccinated can also be vaccinated. Although the vaccine’s effectiveness in adults over 26 years of age is somewhat controversial, recent studies have reported that the vaccine is also effective in adults, especially in adults with little or no sexual experience. 

Currently, in Uzbekistan, a national HPV vaccination program has been implemented since 2019. The primary target cohort is 9 years old, and so far, more than 90% of girls have been successfully vaccinated [38]. Since it takes several decades for cervical cancer to develop, it is possible to verify the vaccine’s effectiveness by comparing the incidence of cervical cancer in the population before and after vaccination in the long term. Although the age at which vaccination is most effective is less than 12 years, it may be necessary to consider vaccination in adults between the ages of 13 and 26.

### 3.4. Benefits of Early Screening and Examples in Korea

The most commonly used methods for early detection of cervical cancer are Pap smear cytology and liquid-based cytology. In a Pap smear, cells collected from the cervix are smeared on a slide, and the brush is discarded, whereas in liquid-based cytology the brush is immersed in liquid to perform cytology. Therefore, the examination form is similar to the examinee’s point of view. Although the accuracy of the two tests slightly differs between studies, it is generally considered similar [48]. However, there were studies that liquid-based cytology had a lower ratio of unsatisfactory samples than a Pap smear. For example, Siebers et al. [49] reported that the unsatisfactory sample rate was 0.37% for liquid cytology and 1.09% for cervical cytology, and the New Technologies for Cervical Cancer Screening Working Group [50] reported that the corresponding value of liquid cytology was 2.6% and cervical cytology was 4.1%.

Recently, attempts have been made to increase the efficiency of the HPV virus test alone or in combination with the cytology test. In the NTCC phase 2 study, no significant reduction in cervical cancer incidence was found compared to cervical cytology (RR: 0.8, *p* = 0.74) [48,51]. As a result of a meta-analysis of European randomized studies, the combined HPV test and cytology significantly reduced the incidence of cervical cancer compared to cytology alone (RR: 0.52, *p* = 0.04) [48]. However, the combined test increased false-positive results, consuming health resources and causing psychological stress to the examinees. In addition, in resource-limited settings, it is difficult for HPV testing to be widely implemented for economic reasons [52].

Currently, Korean cancer screening recommendations suggest a cytology test once a year for all women who have had sexual experience or are over 20 years old [48]. In South Korea, the incidence of cervical cancer has continued to decrease, and the 5-year survival rate of cervical cancer reached 80% in 2015–2019 [5]. The implementation of nationwide cytology-based screening has shown great health benefits in South Korea. Cho [53] reported that cervical cancer screening reduces cervical cancer mortality by as much as 64% in Korea. Of note, when the number of screenings was 1, 2, 3 or more, the mortality rate of cervical cancer decreased by 59%, 77%, and 87%, respectively; therefore, the effectiveness of continuous screening was observed. A prospective cohort study in the UK also reported that cervical cancer screening could reduce the incidence of cervical cancer by 62% [54].

According to the 2020 cancer country profile, there is no cervical cancer screening program in Uzbekistan yet [10]. The easiest method to apply is screening based on visual inspection. In a study by Sankaranarayanan et al. [55] 114 regions of India were randomly assigned, and visual inspection with 4% acetic acid by trained nurses was performed in 57 regions and the other 57 regions were studied as a control group. Cervical cancer was reduced by 25%, and mortality from cervical cancer was reduced by 35%. In particular, it has been reported that the screening was most effective between the ages of 30 and 39 years. Cytology screening-based cervical cancer screening requires trained professionals and laboratory settings, but since the benefits of screening are well known, it should be urgently planned and performed. HPV testing can be selectively considered for high-risk groups considering socio-economic costs.

### 3.5. Advice on Treatment

Early cancer (stage IA or lower) can be treated surgically via conization or hysterectomy. Regarding intermediate-stage cancer (IB-IVA), radiation therapy could be preferred as it yields fewer complications with similar survival outcomes [56,57]. In Uzbekistan, most mid-stage cancers are treated surgically due to the scarcity of radiotherapy equipment and specialists [12]. Radiation therapy has the advantage that additional costs are small when equipment is completed. Therefore, it is recommended to establish the latest radiation equipment (including CT planner, megavoltage LINAC) and train manpower in radiation oncology. Regarding systemic treatment, immunotherapy has recently obtained effective results, but classical drugs such as cisplatin and paclitaxel are the main basis for the treatment of cervical cancer [57]. These drugs have been developed for decades and have the advantage of being relatively inexpensive.

## 4. Conclusions and Future Directions

Cancer incidence in countries increases as HDI increases, as in Uzbekistan. From a global perspective, cancer incidence is in the middle range. However, the mortality is significant with a high mortality per incidence ratio. In Uzbekistan, the three major cancers—breast, stomach, and lung cancer—account for about one-third of cancer incidence and mortality. Accompanying cervical cancer, colorectal cancer, and liver cancer, six major cancers accounts for a little more than half of cancer incidence and mortality.

We present the following recommendations for future cancer prevention and treatment strategies of Uzbekistan. The suggestion is classified as a strong recommendation if the impact is significant and the possibility of implementation is high. The suggestion is classified as a recommendation if it has a significant impact but a moderate feasibility. Even if the impact of the suggestion is significant, it is classified as a recommendation if it requires a mid- to long-term plan or if it is an academic proposal.

Although breast cancer is a female cancer, it is the one of the leading causes of cancer death in Uzbekistan. Compared with Korea, the incidence of breast cancer is about 40%, but the mortality is double (incidence per mortality, 48.5% vs. 10%). Self- of periodical clinical examination and national mammography screening are necessary for the early detection of breast cancer (strongly recommended). The cause of breast cancer needs to be investigated, and radiation therapy and breast-conserving surgery should be more widely performed (recommended). Economic consideration at the government level is necessary to provide costly drugs of targeted therapy or hormone therapy.

Although cervical cancer is a female cancer, it has a prevalence of 6.4% in Uzbekistan. Many patients are diagnosed at an advanced stage, and the mortality rate is high, necessitating early diagnosis with screening. A successful vaccination program performance in Uzbekistan should be commended. Nationwide regular cytology or visual inspection screening is highly necessary (strongly recommended). Radiation therapy facilities should be expanded to provide treatment with fewer side effects for middle-aged patients (recommended). Education on HPV and cervical cancer is necessary so that people do not hesitate to consult the disease or undergo screening.

## 5. Limitations

Despite rigorous searching, the domestic literature on the causes and treatment of cancer in Uzbekistan was very scarce. Therefore, we had to largely base our research on reports published by international organizations such as the WHO and IARC. The data of these reports are mostly descriptive statistics and lack detailed clinical content. This study covers the wide range of information on the prevention and treatment of breast and cervical cancer. However, this study has limitations in terms of discussing in-depth cancer biology such as the tumor microenvironment and cancer genetics, and the development of treatment accordingly.

## Figures and Tables

**Table 1:** Brief global statics of cancer, including Uzbekistan and South Korea.

	Uzbekistan	South Korea	South-Central Asia	Sub-Saharan African Regions	East Asia	Western Europe	Northern America	Australia and New Zealand
Incidence	108.1	242.7	102.5	111.1–130.2	217.2	325	360.7	447.6
Mortality	72.4	75.5	67	78.4–90.1	123.2	103.3	87.1	85.8
Incidence per mortality	67.0%	31.1%	65.4%	69.2–70.6%	56.7%	31.8%	24.1%	19.2%

All values are age-standardized rates per 100,000 population. Data Source: incidence and mortality are from GLOBOCAN 2020 [6,7,8,9].

**Table 2:** Brief global statics of breast cancer, including Uzbekistan and South Korea.

	Uzbekistan	South Korea	South-Central Asia	Sub-Saharan African Regions	Eastern Asia	Western Europe	Northern America	Australia and New Zealand
Incidence	26.4	64.2	26	32.7–39.5	43.3	90.7	89.4	95.5
Mortality	12.8	6.4	13	10.4–18	9.8	15.6	12.5	12.1
Incidence per mortality	48.5%	10.0%	50.0%	31.8–45.6%	22.6%	17.2%	14.0%	12.7%

All values are age-standardized rates per 100,000 population. Data Source: incidence and mortality are from GLOBOCAN 2020 [6,7,8,9].

**Table 3:** Brief global statics of cervical cancer, including Uzbekistan and South Korea.

	Uzbekistan	South Korea	South-Central Asia	Sub-Saharan African Regions	Eastern Asia	Western Europe	Northern America	Australia and New Zealand
Incidence	11	8.1	15.3	22.9–40.1	10.8	7	6.1	5.6
Mortality	6.7	1.8	9.6	16.6–28.6	4.9	2	2.1	1.6
Incidence per mortality	60.9%	22.2%	62.7%	71.3–72.5%	45.4%	28.6%	34.4%	28.6%

All values are age-standardized rates per 100,000 population. Data Source: incidence and mortality are from GLOBOCAN 2020 [6,7,8,9].
